# Prone positioning in ARDS patients supported with VV ECMO, what we should explore?

**DOI:** 10.1186/s40560-022-00640-5

**Published:** 2022-10-04

**Authors:** Hongling Zhang, Zhengdong Liu, Huaqing Shu, Yuan Yu, Xiaobo Yang, Ruiting Li, Jiqian Xu, Xiaojing Zou, You Shang

**Affiliations:** 1 Department of Critical Care Medicine, Union Hospital, Tongji Medical College, Huazhong University of Science and Technology, Wuhan, 430022 China; 2 Department of Intensive Care Unit, Affiliated Lu’an Hospital, Anhui Medical University, Lu’an, 237000 China

**Keywords:** ARDS, VV ECMO, Prone positioning, Effects, Individualized therapy, Computed tomography, Lung ultrasound, Electrical impedance tomography

## Abstract

**Background:**

Acute respiratory distress syndrome (ARDS), a prevalent cause of admittance to intensive care units, is associated with high mortality. Prone positioning has been proven to improve the outcomes of moderate to severe ARDS patients owing to its physiological effects. Venovenous extracorporeal membrane oxygenation (VV ECMO) will be considered in patients with severe hypoxemia. However, for patients with severe hypoxemia supported with VV ECMO, the potential effects and optimal strategies of prone positioning remain unclear. This review aimed to present these controversial questions and highlight directions for future research.

**Main body:**

The clinically significant benefit of prone positioning and early VV ECMO alone was confirmed in patients with severe ARDS. However, a number of questions regarding the combination of VV ECMO and prone positioning remain unanswered. We discussed the potential effects of prone positioning on gas exchange, respiratory mechanics, hemodynamics, and outcomes. Strategies to achieve optimal outcomes, including indications, timing, duration, and frequency of prone positioning, as well as the management of respiratory drive during prone positioning sessions in ARDS patients receiving VV ECMO, are challenging and controversial. Additionally, whether and how to implement prone positioning according to ARDS phenotypes should be evaluated. Lung morphology monitored by computed tomography, lung ultrasound, or electrical impedance tomography might be a potential indication to make an individualized plan for prone positioning therapy in patients supported with VV ECMO.

**Conclusion:**

For patients with ARDS supported with VV ECMO, the potential effects of prone positioning have yet to be clarified. Ensuring an optimal strategy, especially an individualized plan for prone positioning therapy during VV ECMO, is particularly challenging and requires further research.

## Introduction

Acute respiratory distress syndrome (ARDS) is defined by the association of hypoxemia and bilateral lung infiltrates that result from alterations in alveolar-capillary permeability [1]. ARDS appears to be an important global public health issue, especially in the context of the current coronavirus disease 2019 (COVID-19) pandemic [2]. This syndrome is life-threatening and associated with a high mortality rate, particularly for those with moderate and severe ARDS (40.3% and 46.1% of all cases, respectively) [3].

Prone positioning (PP) has been proved to reduce mortality in patients with moderate to severe ARDS [4, 5]. When patients with refractory hypoxemia or hypercapnia continue to deteriorate despite proven management strategies or are unable to maintain protective ventilation, early venovenous (VV) extracorporeal membrane oxygenation (ECMO) has been shown to have a clinically significant benefit [6]. Recently, an increasing number of researchers have expressed interest in the combination of PP and VV ECMO, showing that PP maneuvers during VV ECMO are safe and that complications such as minor hemorrhages at the site of cannula insertion and ECMO flow instability are controllable [7–9]. However, these studies were observational, retrospective, and yielded contradictory results, so the potential benefit of PP maneuvers during VV ECMO remains uncertain and will be discussed in this review. Furthermore, some controversial issues, such as indications, optimal timing, duration, and frequency of PP, as well as the management of respiratory drive, will also be discussed.

## Potential effects of prone positioning during VV ECMO

### Gas exchange

It is well known that PP can improve oxygenation in patients with ARDS owing to a reduction in ventilation-perfusion and shunt heterogeneity, while the role of PP remains unclear when combined with VV ECMO. Several preliminary observational studies and meta-analyses have reported that performing PP during ECMO for severe ARDS is safe, and PP could improve the partial pressure of arterial oxygen to the fraction of inspired oxygen (PaO_2_/FiO_2_) ratio, consistent with the effects of the duration of PP performed; this improvement was maintained after resupination [8, 10–13]. However, is it important to predict the oxygenation response? When patients with ARDS receive PP without ECMO, gas exchange in relation to PP is determined by the quantity of tissue open to ventilation and perfusion during the respiratory cycle, the degree of homogeneity of inflation, and regional changes in chest wall compliance [9]. A retrospective analysis of data from the PROSEVA study showed that improvement in blood gases during the PP session was not associated with an increase in survival [14]. Moreover, a study of patients with COVID-19-related ARDS supported with ECMO showed a significantly higher PaO_2_/FiO_2_ ratio after PP, while the mortality of the prone group was higher than that of the supine group [10]. Hence, it is likely not a major concern to focus on the oxygenation response to PP during ECMO. Moreover, for severe ARDS patients receiving VV ECMO, arterial oxygenation is mainly determined by ECMO flow/cardiac output [15]; in ARDS patients with preload reserve, the cardiac index may increase during PP mainly resulting from right ventricular unloading [16, 17]. Notably, the combination of PP combined with ECMO is complex; oxygenation could more likely be influenced by ECMO settings than by the effects of PP.

In contrast, Gattinoni et al. suggested that decrease in partial pressure of carbon dioxide (PaCO_2_) (decreased physiologic dead space ratio) with PP is predictive of improved outcome in ARDS [18]. Recently, a study used PaCO_2_ instead of oxygenation as a key marker to identify the effects of PP on ARDS patients receiving VV ECMO; they exhibited a significant decrease in PaCO_2_ from 39 (34–41) mmHg to 31 (29–37) mmHg after a PP session (*P* = 0.03) without any variation in sweep gas flow [19]. Overall, the exact effect of PP on gas exchange in ARDS patients supported on VV ECMO remains uncertain; rather than oxygenation, PaCO_2_ might be an alternative parameter to assess the effect of PP.

### Respiratory mechanics

PP generates a more homogeneous distribution of stress and strain in patients with ARDS. During VV ECMO, the effects of PP on respiratory mechanics are still controversial. Some studies showed that static compliance of the respiratory system significantly improved after the PP cycle during VV ECMO [11, 12], while other studies showed that PP improved oxygenation without a change in respiratory system compliance [10, 13]. Additionally, instead of gas exchange, two studies [19, 20] used static compliance to evaluate PP response by analyzing the impact of PP on respiratory mechanics. After 16 h of PP during ECMO, an increase in static compliance greater than or equal to 3 mL/cm H_2_O compared to baseline was defined as PP responders. However, in these studies, the proportion of PP responders was 53% [20] and 62% [19], respectively. Moreover, PP responders had a significantly higher probability of weaning from ECMO and remaining alive within 90 days, shorter ECMO duration and shorter intensive care unit (ICU) length of stay, and their 90-day mortality tended to be lower (15% vs. 23%) [19, 20]. Franchineau et al. showed that PP responders had a higher body mass index, more frequent occurrence of viral pneumonia, shorter ECMO duration, and lower baseline tidal volume (VT) (dorsal)/VT(global) ratio (measured by electrical impedance tomography (EIT)) than patients with PP non-responders [19], but the difference was absent in the more recent study by Petit et al. [20].

Patients on VV ECMO frequently require ultra-protective ventilation with a very low tidal volume, which can result in pulmonary derecruitment if positive end-expiratory pressure (PEEP) is not properly titrated. Unfortunately, there is currently no precise method for configuring PEEP, such as esophageal pressure-guided PEEP [21], especially in severe ARDS patients supported on VV ECMO. EIT has the potential to be an appealing bedside non-invasive method for providing real-time monitoring and setting optimal PEEP [19]. One study that used EIT to titrate PEEP found a wide range of optimal PEEP in severe ARDS patients on VV ECMO, reinforcing the need for individualized ventilation titration [22]. Given the potential impact of PP on respiratory mechanics, a sufficient level of PEEP in the prone and supine positions can be different; hence, optimal PEEP should ideally be tailored to each patient rather than being applied uniformly to all.

### Hemodynamics

In patients with severe ARDS, hypoxemia, hypercapnia, high driving pressure, and high plateau pressure are risk factors for developing acute cor pulmonale, which appears to be the leading cause of death [23–25]. VV ECMO can reverse hypoxemia and acidosis while reducing the burden of mechanical ventilation on the lung to provide ultraprotective ventilation, and PP has the potential to reduce driving pressure and plateau pressure by recruiting the lungs, which is beneficial to right ventricular function and hemodynamics [9, 26]. However, there is limited evidence regarding the effects of using PP on hemodynamics in patients treated with VV ECMO. Two studies showed no significant changes in hemodynamics [27, 28], and one study showed minor differences in hemodynamics (mean pulmonary arterial pressure and pulmonary wedge pressure were slightly higher during PP, and heart rate was lower) [12], while these studies were retrospective with few cases. These effects of PP on hemodynamics during ECMO are hardly predictable, suggesting that careful hemodynamic monitoring is needed. Notably, in patients on VV ECMO, conventional methods such as pulmonary artery thermodilution and transpulmonary thermodilution are known to be inaccurate for cardiac output measurement due to indicator loss into the extracorporeal circuit [29–32]. Transthoracic echocardiography, a bedside noninvasive technique, will play an important role in monitoring cardiac function, and transesophageal echocardiography has been shown to be safe in ARDS patients on VV ECMO despite systemic anticoagulation [33–35].

Additionally, since ARDS is frequently associated with sepsis-induced acute circulatory failure [36], it is important to determine whether ARDS patients with hemodynamic instability can be placed in the prone position. In the PROSEVA trial, which showed a beneficial effect of PP on survival, 72% of patients in the PP group received vasopressors [4]. Recently, a study comparing patients with PP during ECMO and those without PP also showed a significant proportion of patients receiving vasopressors in both the PP group (69%) and the no-PP group (76%) [20]. Receiving vasopressors is not a contraindication to PP if the mean arterial pressure can be maintained at ≥ 65 mmHg. In the case of hypotension, fluid responsiveness should be evaluated prior to PP. Fluid responsiveness predictors are required because volume expansion can aggravate pulmonary edema, and volume overload must be avoided. However, in severe ARDS patients on VV ECMO, dynamic parameters to predict fluid responsiveness, such as pulse pressure variation and stroke volume variation, are limited due to protective ventilation, right-sided heart failure, pulmonary hypertension, and the effects of the ECMO system on heart–lung interaction; in this situation, passive leg raising-induced changes in stroke volume greater than 10% as measured by transthoracic echocardiography may predict fluid responsiveness [37].

### Outcomes

In severe ARDS patients supported on VV ECMO, survival, ECMO duration, mechanical ventilation duration, and ICU length of stay are the most common outcomes that clinicians are concerned about. The mortality of the included patients varies across different centers, resulting from various characteristics of patients, PP strategies and protocols. Some studies showed beneficial outcomes when patients were treated with VV ECMO and PP, while some experienced adverse outcomes (Table 1). Recently, a systematic review and meta-analysis including 11 retrospective studies showed that cumulative survival in patients who underwent PP was higher than that in patients without PP during VV ECMO (57% vs. 47%), but the difference was not statistically significant. Additionally, patients who underwent PP had a longer ICU length of stay and ECMO duration [8]. However, because this meta-analysis was based on aggregate data, it may be skewed by differences in the characteristics of patients treated with PP and controls. In contrast, a recent analysis of pooled individual patient data (total *n* = 889) revealed that the use of PP during ECMO was not associated with lower mortality (ICU mortality: prone group vs. supine group, 39.6% vs. 48%, *P* = 0.072); when patients were matched on baseline characteristics using a propensity score, those in the prone group had a significantly longer ECMO duration and a higher 60-day survival rate [38]. Interestingly, rather than the primary outcome of pooled cumulative survival based on studies reporting varying survival interval data as reported by Poon et al. [8], a systematic review and meta-analysis of 13 studies (total *n* = 1836) by Papazian et al. [39] considered timepoints based on the exact rates obtained from the authors when not reported in the original studies, demonstrating that PP was associated with a significant improvement in 28-, 60-, 90-day and ICU survival (28-day survival: prone group vs. supine group, 74% vs. 58%, *P* < 0.001); furthermore, this distinction was observed in both COVID-19 and non-COVID-19 ECMO patients. However, these findings were limited by the observational nature of the studies and the presence of residual and unmeasured confounders; thus, prospective randomized controlled trials are required to thoroughly evaluate the impact of PP on ARDS patients receiving VV ECMO. One study (ClinicalTrials.gov Identifier: NCT04607551) was registered recently with the objective to analyze whether PP facilitates weaning from ECMO in patients with refractory ARDS.

**Table 1:** Comparison of different studies on VV ECMO combined with prone positioning in patients with severe ARDS

References	Pre ECMO			During ECMO						Mortality (%) PP vs. SP
	Score	Receiving PP, *n* (%)	MV-to-ECMO Day, d	Reasons to perform PP	ECMO-to-PP Day, d	Receiving PP, *n* (%)	PP duration per session, h	PP sessions	Sedation and NMBA	
*Zaaqoq [77] Multicenter, retrospective 2022	SOFA 7 (IQR 4–9)	49 (73)	4 (IQR 2–8)	Depended on clinician discretion	NA	67 (29)	NA	6 (IQR 2–14)	NA	Hospital: 67 vs. 78 (hazard ratio, 0.31; 95% CI 0.14–0.68)
Petit [20] Single center retrospective 2022	SOFA 13 (IQR 9–16)	55 (86)	4 (IQR 1–9)	Severe hypoxemia, extensive lung consolidation, or difficult ECMO-weaning	3 (IQR 2–6)	64 (21)	16	2 (1–2) per patients	Deeply sedated with NMBA	90-day, unadjusted: 20 vs. 48 (*P* < 0.01), adjusted: 20 vs. 42 (*P* < 0.01)
*Laghlam [42] Single center prospective 2021	SOFA 11 (IQR 6–14)	10 (100)	5 (IQR 4–10)	Based on PaO_2_/FiO_2_ ratio value and left at the discretion of the attending physician	2 (IQR 1–3)	10 (41)	17.4 ± 2.1	3 (IQR 2–5)	Deeply sedated with NMBA	60-day, unadjusted: 40 vs. 43 (*P* = 0.99)
Giani [12] Multicenter, retrospective 2021	SOFA 9 ± 3	34 (31)	2 (IQR 1–6)	Based on the clinical judgement of attending physicians	4 (IQR 2–7)	107 (45)	15 (IQR 12–18)	Total 326	NA	Hospital, unadjusted: 34 vs. 49 (*P* = 0.017), adjusted: 30 vs. 53 (*P* = 0.024)
Rilinger [44] Single center, retrospective 2020	SOFA 11 (IQR 11–15)	7 (18)	2.2 (IQR 0.2–7.6)	Judged by the treating medical team	1.7 (IQR 0.5–5.0)	38 (24)	19.5 (IQR 16.8–20.8)	2 (1–3) per patients	Titrated to preserve spontaneous breathing if possible	Hospital, unadjusted: 63.2 vs. 63.3 (*P* = 0.984), adjusted: 63.2 vs. 63.2 (*P* = 1.0)
*Garcia [10] Single center retrospective 2020	SAPS II 59.5 (IQR 46–62)	14 (100)	6.5 (IQR 4–10)	Severe hypoxemia or extensive lung consolidation on chest imaging (> 50% of lung volume)	1.5	14 (56)	16 (IQR 15–17)	Total 24	NA	28-day, unadjusted: 78.6 vs. 27.3 (*P* = 0.02)
Franchineau [19] Single center prospective 2020	SOFA 13 (IQR 11–16)	16 (76)	8 (IQR 6–11)	ARDS patients on VV ECMO without contraindications	2 (IQR 1–5)	21 (100)	16	2 (1–2) per patients	Deeply sedated and paralyzed	NA
Guervilly [41] Single center retrospective 2019	SOFA 10 ± 4	69 (76)	5 ± 5	Persistent hypoxemia, failure of attempt to wean ECMO after at least 10 days of ECMO and the presence of lung consolidations on chest X-ray or lung ultrasounds, or according to the physician in charge of the patient	5 ± 4	91 (54)	12–16	3 (1–17)	Deeply sedated and paralyzed	90-day, unadjusted: 38 vs. 58 (*P* = 0.008), adjusted: 42 vs. 64 (*P* = 0.028)
Kimmoun [11] Single Center, retrospective 2015	SOFA 12 (IQR 8–15)	13 (76)	NA	Refractory hypoxemia combined or not with persistent high plateau pressure or unsuccessful ECMO weaning attempt after day 7	6 (IQR 4–12)	17 (38)	24	Total 27	NA	NA
Guervilly [13] Single center prospective 2014	SOFA 9 (IQR 8–11)	9 (60)	6.5 (IQR 1–9)	Severe hypoxemia, injurious ventilation parameters or failure of attempt to wean ECMO after at least 10 days of ECMO	8 (IQR 5–10)	15 (32)	12	Total 21	Deeply sedated and paralyzed	NA

*NA* not applicable, *SOFA* sequential organ failure assessment, *SAPS II* simplified acute physiology score, *IQR* interquartile range, *CI* confidence interval, *PP* prone positioning, *SP* supine positioning, *VV* venovenous, *ECMO* extracorporeal membrane oxygenation, *MV* mechanical ventilation, *NMBA* neuromuscular blocking agents

^*^Studies focused on patients with SARS-CoV-2-induced ARDS

## Controversial opinions of prone positioning during VV ECMO

### Indications

For patients without ECMO support, international recommendations suggested that PP should be used in adult patients with moderate-severe ARDS [5, 40]. However, for patients supported with VV ECMO, the indications for PP remain uncertain. Several retrospective studies utilized PP as a rescue therapy for severe ARDS patients supported with VV ECMO, such as severe hypoxemia, extensive lung consolidation on chest imaging and unsuccessful ECMO weaning [10, 11, 20, 41, 42]. However, a recent monocentric study found that at the end of PP, all patients supported with VV ECMO had a decrease in EIT-estimated optimal PEEP and an improvement in local compliance, VT, and end-expiratory lung impedance redistribution, highlighting the potential reduction of atelectasis with PP; consequently, the authors suggested to prone all ARDS patients supported with VV ECMO regardless of their predicted response in terms of static compliance improvement [19].

The use of ultraprotective ventilation after VV ECMO implantation may result in derecruitment. PP can help maintain lung recruitment and facilitate secretion drainage. Owing to this physiological rationale, combining PP and VV ECMO may benefit all patients with severe ARDS, indicating that PP could be a routine therapy during ECMO support. However, given the scarcity of available data, the evidence-based recommendation is lacked. In addition, PP during ECMO is a difficult procedure that may be associated with additional complications when performed in ECMO centers lacking specific expertise. Furthermore, the PP maneuver requires four to six health care providers, which may be challenging, particularly in COVID-19 ARDS patients. As a result, it is critical to determine who will benefit from PP during ECMO support. Patients on ECMO who are still at risk of ventilator-induced lung injury may be an indication to implement PP, which requires further research.

### Timing

The optimal timing of PP during VV ECMO is still unclear. Two studies have compared the effects of different timings of PP during VV ECMO. Kimmoun et al. showed that the improvement of oxygenation appeared to be more efficient when PP was implemented after 7 days of VV ECMO. In their opinion, time is a significant factor in allowing symptomatic treatments to be effective and lung healing; equally, lung injury decreases owing to ultraprotective ventilation during VV ECMO [11]. However, it has been proposed that improvement in oxygenation is not associated with survival benefit; we need to consider the outcome instead of the improvement of oxygenation [43]. In contrast, a single-center retrospective study reported that early initiation of PP was linked to a significant reduction in hospital mortality, and the survival of patients treated with early PP (cutoff < 17 h via Youden’s Index) was higher than that of patients treated with late PP or without PP during VV ECMO support (81.8% vs. 33.3%). The vasoactive support ratio, SOFA scores, and APACHE II scores at the time of VV ECMO implantation were not different between these two groups, whereas patients in the early PP group were younger than patients in the late PP group or without PP, which could be a bias [44]. Hence, owing to this controversial topic, large randomized controlled trials regarding the timing of PP with ECMO are needed. One study (ClinicalTrials.gov Identifier: NCT04139733) aiming to evaluate whether early PP could reduce VV ECMO duration is ongoing.

### Duration and sessions

PP is highly advocated in patients with moderate-severe ARDS, while the optimal duration of PP sessions is not definitively determined. The PROSEVA study found that in ARDS patients with a ratio of PaO_2_/FiO_2_ < 150 mmHg, the use of PP for at least 16 h a day reduced 90-day mortality from 41.0 to 23.6% (*P* < 0.001), with no obvious adverse effects [4]. Subsequently, clinical practice guidelines strongly recommend that adult patients with moderate-severe ARDS receive PP for more than 12–16 h per day [5, 40]. Recently, a study suggested that PP sessions should be extended to at least 24 h and should be prolonged when the PaO_2_/FiO_2_ ratio at 24 h remains below 150 mmHg [45]. It is worth noting that we should stop PP in the event of deterioration of oxygenation or a life-threatening complication during PP. In the existing studies of ECMO combined with PP, most researchers employed PP for approximately 16 h per session during VV ECMO (Table 1). One study found that extending PP to 24 h per session in ARDS patients on VV ECMO improved oxygenation and respiratory system compliance without major adverse events; however, this study did not compare the outcomes of the PP and non-PP groups [11].

In addition, the optimal frequency of PP during VV ECMO have not been determined. Different studies have reported different PP sessions (Table 1). Premature judgment of no response to PP should be avoided. PP may be ineffective in the early stage due to the typically severe pulmonary inflammation at this time. In the PROSEVA trial, PP was repeated until the patients’ oxygenation improved. The average number of sessions was four, with PP still effective from the third to fifth [4]. Moreover, methods for assessing the effectiveness of PP are urgently needed. Without evaluation, sometimes PP is ineffective but continues to be implemented, increasing the workload of medical staff and the likelihood of complications developing. In future clinical trials, consideration of duration and sessions of PP is essential.

### Respiratory drive

In the majority of studies, ARDS patients receiving VV ECMO were deeply sedated and paralyzed during PP sessions, primarily owing to concerns about high respiratory drive and the displacement or malfunction of ECMO circuitry [10, 19, 20]. High respiratory drive should be limited due to the risks of ventilator-induced lung injury, patient self-inflicted lung injury, and diaphragm damage [46]. Excessively low respiratory drive, on the other hand, can result in diaphragm atrophy, whereas spontaneous breathing can preserve diaphragm activity, recruit dependent lung regions, and improve cardiovascular function [47, 48]. In one study, sedation was titrated to preserve spontaneous breathing in patients requiring VV ECMO during PP sessions, and neuromuscular blockade agents was not used routinely except in cases of high respiratory drive. Interestingly, a lower proportion of spontaneous breathing was found to be strongly associated with mortality; however, the evidence is insufficient because low respiratory drive may be an expression of less disease severity [44].

During PP sessions, the optimal target range for respiratory drive in ARDS patients receiving VV ECMO is uncertain. Although it is recommended to avoid excessive respiratory drive, we believe that individualized respiratory drive manipulation is necessary and inseparable from close monitoring. Available bedside monitoring tools for respiratory drive include the methods to monitor neural output (diaphragm electrical activity, electromyography), breathing effort (airway occlusion pressure, airway pressure deflection generated by patient inspiratory effort during an end-expiratory airway occlusion, respiratory muscle pressure, dyspnea, esophageal pressure swings, use of accessory inspiratory and expiratory muscles) and ventilatory response (respiratory rate, rapid shallow breathing index, mean inspiratory flow) [49]. Owing to the absence of the “gold standard” for the clinical assessment of respiratory drive, multimodal evaluation may be the most appropriate method.

## Promising methods for individualized therapy

ARDS is a syndrome including heterogeneous phenotypes with variable clinical and outcome characteristics, and PP is not effective in every case owing to the heterogeneity of ARDS, which highlights the necessity of further studies of the predictors of PP effectiveness. Until now, no criteria have predicted which patient will benefit from PP. Oxygenation and respiratory parameters failed to predict the response to PP [43, 45], while anatomical alveolar recruitability may be an available parameter to evaluate the effects of PP. Lung morphology can be used to distinguish between different types of ARDS, and patients with different lung morphologies are associated with differences in lung mechanics and outcomes [50–52]. Some studies demonstrate the importance of integrating lung morphology and ARDS management [53–55]. In patients with ARDS supported with VV ECMO, computed tomography (CT), lung ultrasound (LUS), and EIT may help clinicians to better determine individualized PP therapy, including assessment of the initial lung morphology before PP, assessment of lung recruitability, and identification of the responders and non-responders to PP.

### Lung computed tomography scan

Lung CT scans are the gold standard imaging technique to assess the distribution of lung strain and quantify the loss of lung aeration in patients with ARDS [56]. Before PP, initial lung morphology may indicate which will benefit more from PP. Patients with typical ARDS can be divided into two phenotypes by lung CT scan: one is focal (lobar loss of lung aeration), and the other is non-focal (diffuse or patchy loss of lung aeration). Patients with focal ARDS are at high risk of hyperinflation during recruitment maneuvers and high PEEP, which may benefit from the PP to gain a more homogenous stress and strain redistribution [54, 56]. Because ARDS patients supported with VV ECMO are unable to transferred due to their critical condition, CT scans cannot be easily performed and repeated to guide the application of PP.

Quantitative lung CT scans were used in two studies to assess the correlation with the effect of PP in severe ARDS patients supported with ECMO. In one study, CT was implemented 1–2 days before the first PP session to assess PP response during VV ECMO. In their study, the lungs were divided into four anatomical regions: ventral, medial-ventral, medial-dorsal, and dorsal. Hounsfield units were then used to identify four lung compartments: hyperinflated, normally aerated, poorly aerated, and nonaerated [57]. Interestingly, the authors found that patients with better normally aerated lung tissue in the ventral and medial-ventral regions before PP were more likely to improve their static compliance after that procedure during ECMO [20]. However, in another study, no correlation was found between the amount of nonaerated lung tissue measured on lung CT scans and the improvement of oxygenation after PP [11]. Nevertheless, neither paper studied the relationship between morphology monitored by lung CT scans and outcomes in prone-positioned patients with ARDS during ECMO, which is of greater concern and needs further study.

### Lung ultrasound

LUS is an attractive tool that can be utilized for bedside assessment of lung morphology and in lung aeration changes [58–61]. To date, there are no published articles using LUS to assess the effects of PP in patients with severe ARDS supported on ECMO. Several studies have investigated whether LUS predicts the response to PP in ARDS patients not on ECMO, which can provide insights for further research.

Before PP, LUS was shown to be a reliable tool to distinguish focal from non-focal morphologies; an LUS score of the ventral fields ≥ 3 was confirmed to be highly predictive of non-focal ARDS morphology, with a sensitivity and specificity of 94% and 100%, respectively, compared with the gold standard CT [62]. Patients with focal ARDS may obtain more benefits resulting from PP [54]. Since CT scans are impractical when patients are unable to be transferred due to illness, especially when supported with ECMO, LUS could be a useful tool for further personalizing the use of PP during ECMO. Throughout and after PP sessions, the ability of LUS score variations can be used to predict the effects of PP [63–67]. However, LUS cannot be used to detect lung overinflation. The feasibility of LUS score variations throughout PP sessions to identify responders and non-responders to PP requires further study.

### Electrical impedance tomography

EIT, an appealing noninvasive, bedside, radiation-free technique, represents a new direction in medical imaging technology [68]. In experimental studies, for mechanically ventilated patients with ARDS, EIT can provide information on alveolar overdistension and assessment of lung recruitability, assess unmatched ventilation and perfusion, individualize ventilation settings (PEEP and tidal volume), and monitor the impact of PP on regional ventilation and optimal PEEP at the bedside [19, 69–74]. However, there is a scarcity of studies on the effect of PP monitored by EIT in severe ARDS patients supported on VV ECMO. Only in one study EIT was utilized to describe the impact of PP on global and regional ventilation in severe ARDS patients supported on ECMO. The feasibility of EIT in such patients was demonstrated. Additionally, through EIT, the authors found that all patients presented an increase in the local compliance and the VT (dorsal)/VT (global) ratio, and EIT-estimated optimal PEEP decreased with PP [19]. We should focus on ventilation-perfusion matching rather than just ventilation. Recently, a study used saline contrast EIT to monitor the physiologic effects of PP in COVID-19-associated ARDS patients, revealing that PP increased lung recruitment, decreased atelectrauma, and improved ventilation–perfusion matching [75]. Unfortunately, the feasibility of saline contrast EIT in patients supported on VV ECMO remains uncertain.

Before PP sessions, EIT can be used to predict and assess lung recruitability. Recently, one study found that dependent lung area collapse (> 13.5%) had an excellent positive predictive value (94%) of improved oxygenation during prone ventilation when monitored by EIT, providing a direction for the individualized treatment of PP; however, the findings need to be confirmed further because this study only included COVID-19-associated ARDS patients and had a small sample size [76]. During and after PP sessions, we can use EIT to estimate ideal PEEP, responders and nonresponders to PP, and the optimal PP duration. More proof is required to support this new method.

## Conclusion

**Fig. 1:**
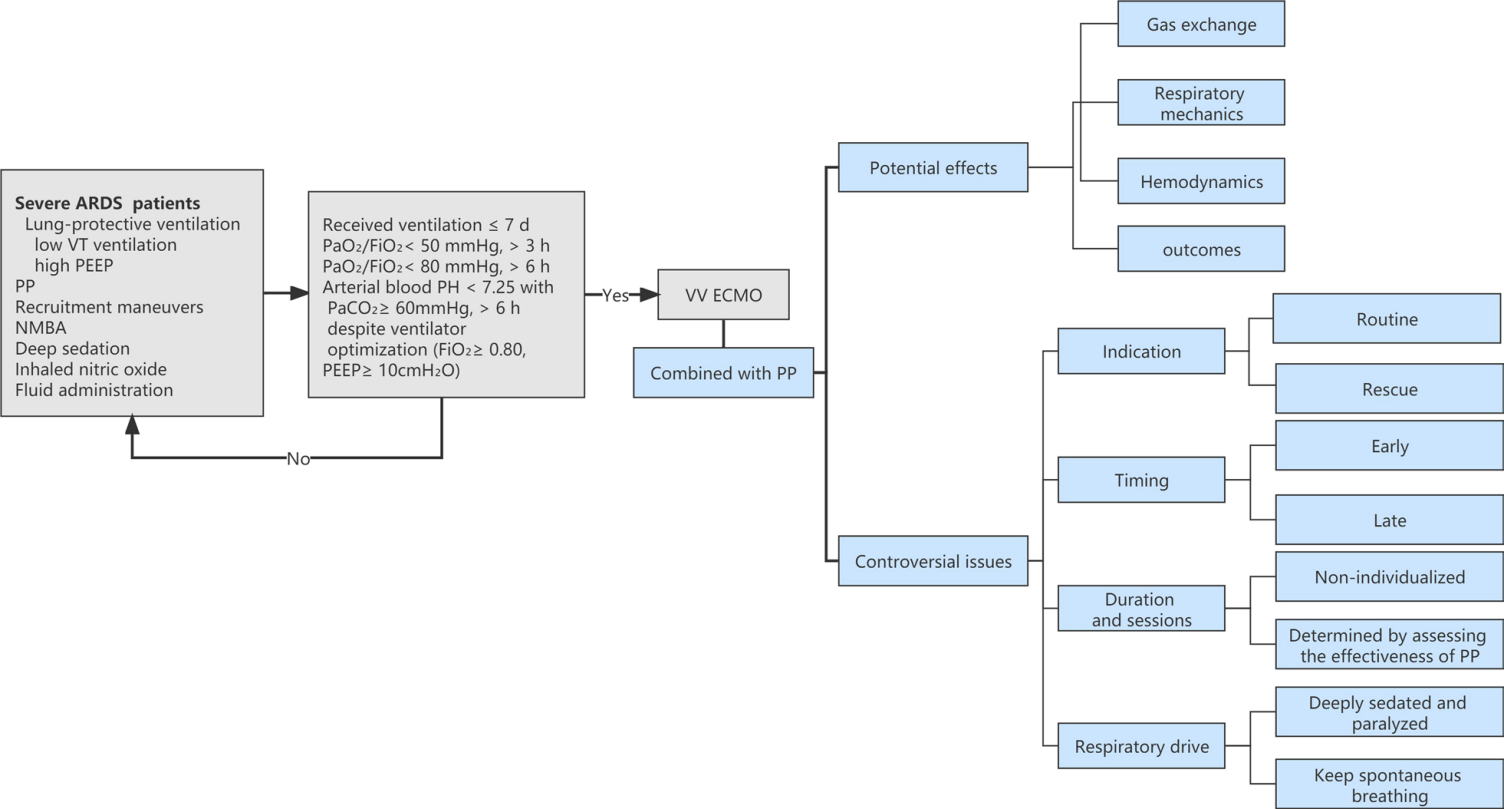
Areas of consensus and controversy in severe ARDS management. Gray box shows the areas of consensus. Blue boxes show areas of controversy and new directions. *ARDS* acute respiratory distress syndrome, *VT* tidal volume, *PEEP* positive-end expiratory pressure, *PP* prone positioning, *NMBA* neuromuscular blocking agent, *FiO*_*2*_ fraction of inspired oxygen, *PaO*_2_ partial pressure of oxygen, *PaCO*_2_ partial pressure of carbon dioxide, *VV ECMO* venovenous extracorporeal membrane oxygenation

The potential effects of PP on gas exchange, respiratory mechanics, hemodynamics, and outcomes in ARDS patients supported with VV ECMO are not definitively determined. Furthermore, a number of questions remain unanswered regarding the combination of PP and VV ECMO, including indications, optimal timing, duration, and sessions of PP, as well as the management of respiratory drive. Ensuring an optimal strategy of PP during VV ECMO is particularly challenging and requires further research (Fig. 1). Lung morphology monitored by CT, LUS, or EIT might be a potential indication for individualized PP therapy in ARDS patients supported with VV ECMO.

## Data Availability

Not applicable.

## Abbreviations

ARDS: Acute respiratory distress syndrome
COVID-19: Coronavirus disease 2019
PP: Prone positioning
VV: Venovenous
ECMO: Extracorporeal membrane oxygenation
PaO_2_/FiO_2_: Partial pressure of arterial oxygen to the fraction of inspired oxygen
PaCO_2_: Partial pressure of carbon dioxide
VT: Tidal volume
EIT: Electrical impedance tomography
PEEP: Positive end-expiratory pressure
ICU: Intensive care unit
CT: Computed tomography
LUS: Lung ultrasound
